# Supplementary material for: Cytokine-armed pyroptosis induces antitumor immunity against diverse types of tumors
Source: Nat Commun. 2024 Dec 30;15:10801. doi: 10.1038/s41467-024-55083-3 (PMC11686184; doi:10.1038/s41467-024-55083-3)
Supplement: Supplementary file 1 — Supplementary Information [file 41467_2024_55083_MOESM1_ESM.pdf]

Supplementary Informations

Cytokine-armed pyroptosis induces antitumor immunity against diverse types of tumors

Sara Orehek[1,2], Taja Železnik Ramuta[1], Duško Lainšček[1,3,4], Špela Malenšek[1,2], Martin Šala[5], Mojca Benčina[1,4,6], Roman Jerala[1,3,4], Iva Hafner-Bratkovič[1,3,7 *]

[1]Department of Synthetic Biology and Immunology, National Institute of Chemistry, Ljubljana, Slovenia.

[2]Interdisciplinary Doctoral Study of Biomedicine, Faculty of Medicine, University of Ljubljana, Ljubljana, Slovenia.

[3]EN-FIST Centre of Excellence, Ljubljana, SI-1000, Slovenia.

[4]Centre for the Technologies of Gene and Cell Therapy, National Institute of Chemistry, Ljubljana, Slovenia.

[5]Department of Analytical Chemistry, National Institute of Chemistry, Ljubljana, Slovenia.

[6]Biotechnical Faculty, University of Ljubljana, Ljubljana, Slovenia.

[7]Faculty of Medicine, University of Ljubljana, Ljubljana, Slovenia.

*Correspondence: iva.hafner@ki.si

Supplementary Figures 1-16 and Tables 1-2

a

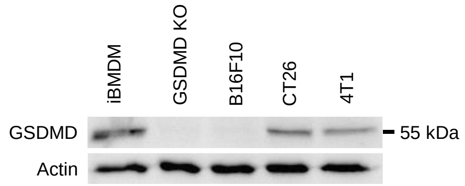

b

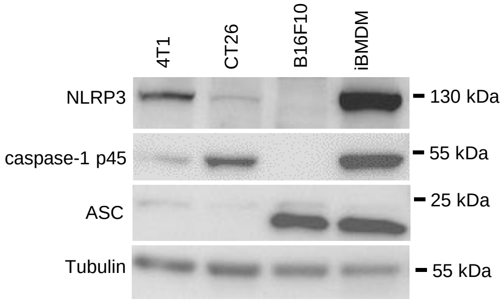

Supplementary Figure 1: Cancer cell lines suppress the expression of inflammasome-related proteins. Expression of endogenous GSDMD (**a**) and NLRP3 inflammasome components (**b**) in B16F10, CT26, and 4T1 cell lines. Representative blots (a, b) of three independent experiments are shown.

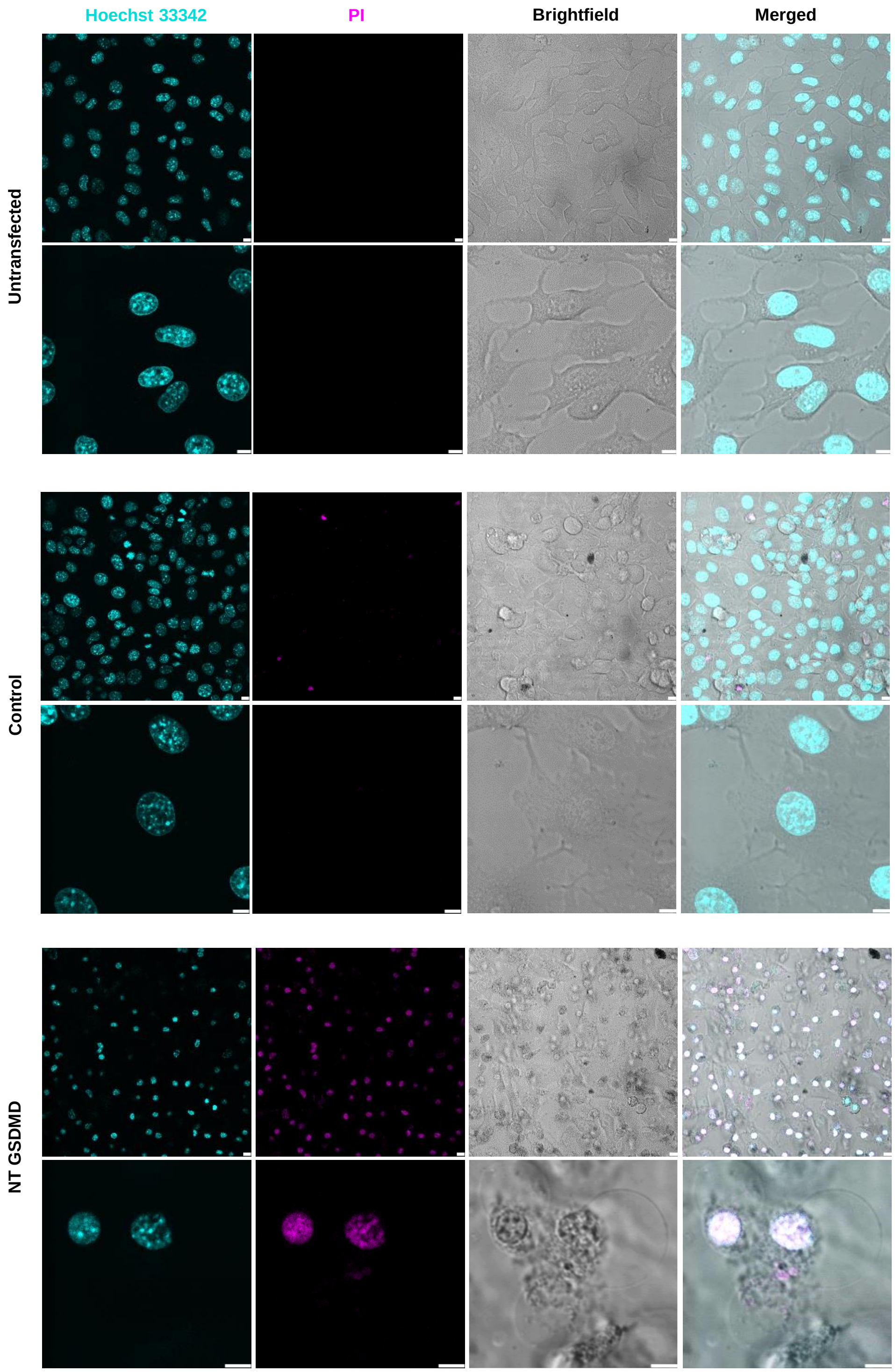

Supplementary Figure 2: NT GSDMD-induced ballooning of B16F10 melanoma cells. Confocal images of NT GSDMD- or empty vector-transfected B16F10 cells stained with Hoechst 33342 (cyan) and PI (magenta). Scale bars 5 µm.

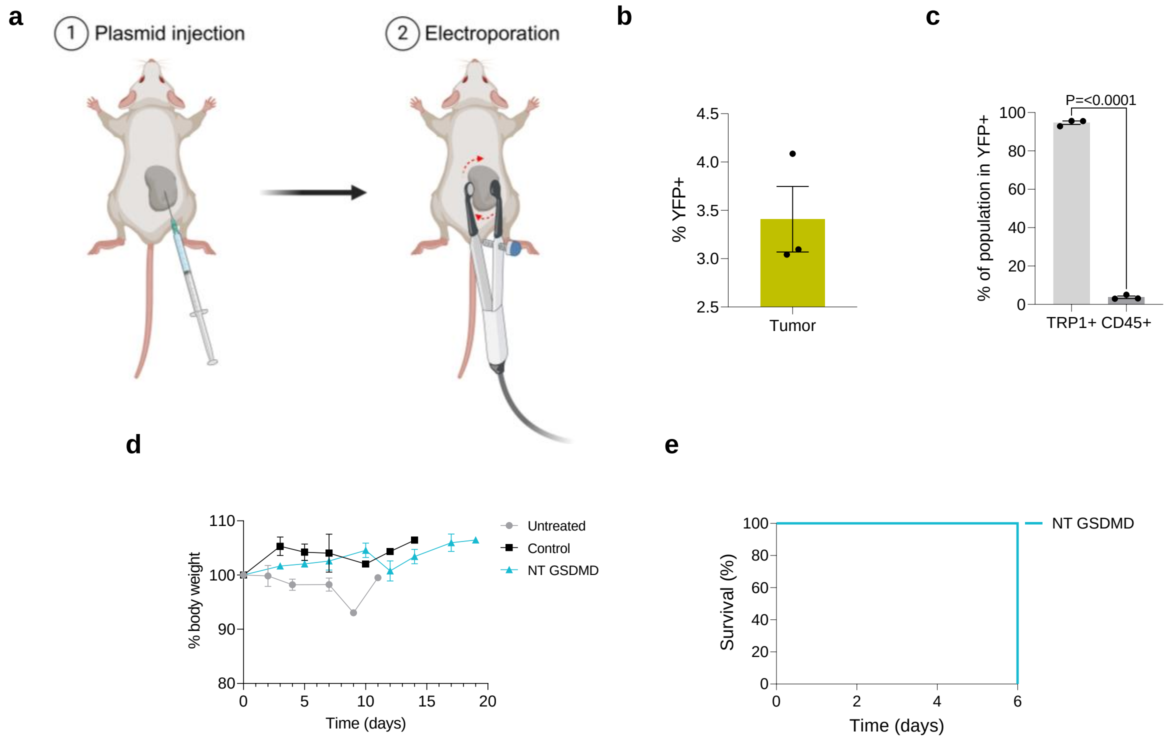

Supplementary Figure 3: Electrogenic transfer. a Schematic of the electroporation procedure. **b** % of YFP-expressing cells in the tumor. **c** % of TRP1+ (melanoma marker) and CD45+ populations among YFP+ cell population. **d** Normalized (%) body weight of representative mice followed in the NT GSDMD experiment. The body weight of individual mice was normalized to the measurement on the day of the first electroporation (day 0). **e** To assess the capacity of plasmid gymnosis, mice were treated according to a standard protocol but unaccompanied by electroporation. Kaplan-Meier curve depicting survival of treated mice (n=3). Plots (**b**, **c**) represent mean ± SEM (n=3) and significance (**c**) was assessed using an unpaired two-tailed t-test. Data (**d**) are shown as mean ± SEM, n=16 for NT GSDMD, n=16 for the control and n=5 for the untreated group. Time (**d, e**) is defined as days post NT GSDMD i.t injection. Source data are provided as a Source Data file. Supplementary Fig. 3a created in BioRender. Hafner Bratkovic, I. (2024) https://BioRender.com/c06a047.

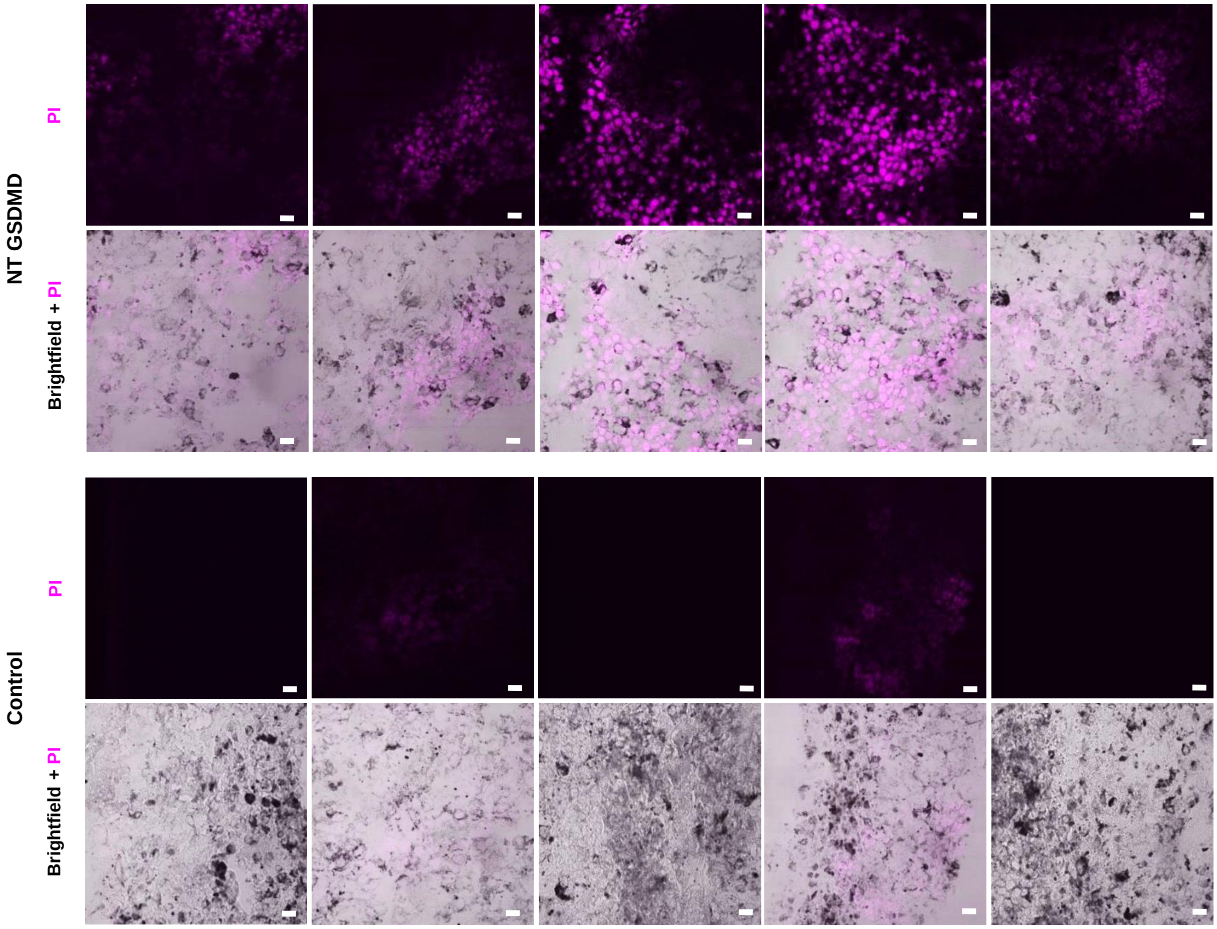

b

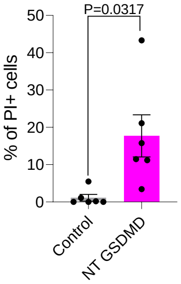

Supplementary Figure 4: In vivo PI labeling after electrogenic transfer of plasmid encoding NT GSDMD or empty vector. **a** Tumor tissue sections after the treatment with NT GSDMD and i.t. PI injection. Scale bars 20 µm. Some images correspond to the same tumor samples as in Fig. 2g. **b** Quantification of PI %. Data (**b**) represents mean ± SEM (n=6) and an unpaired two-tailed t-test with Welch's correction was used for statistical assessment. Source data are provided as a Source Data file.

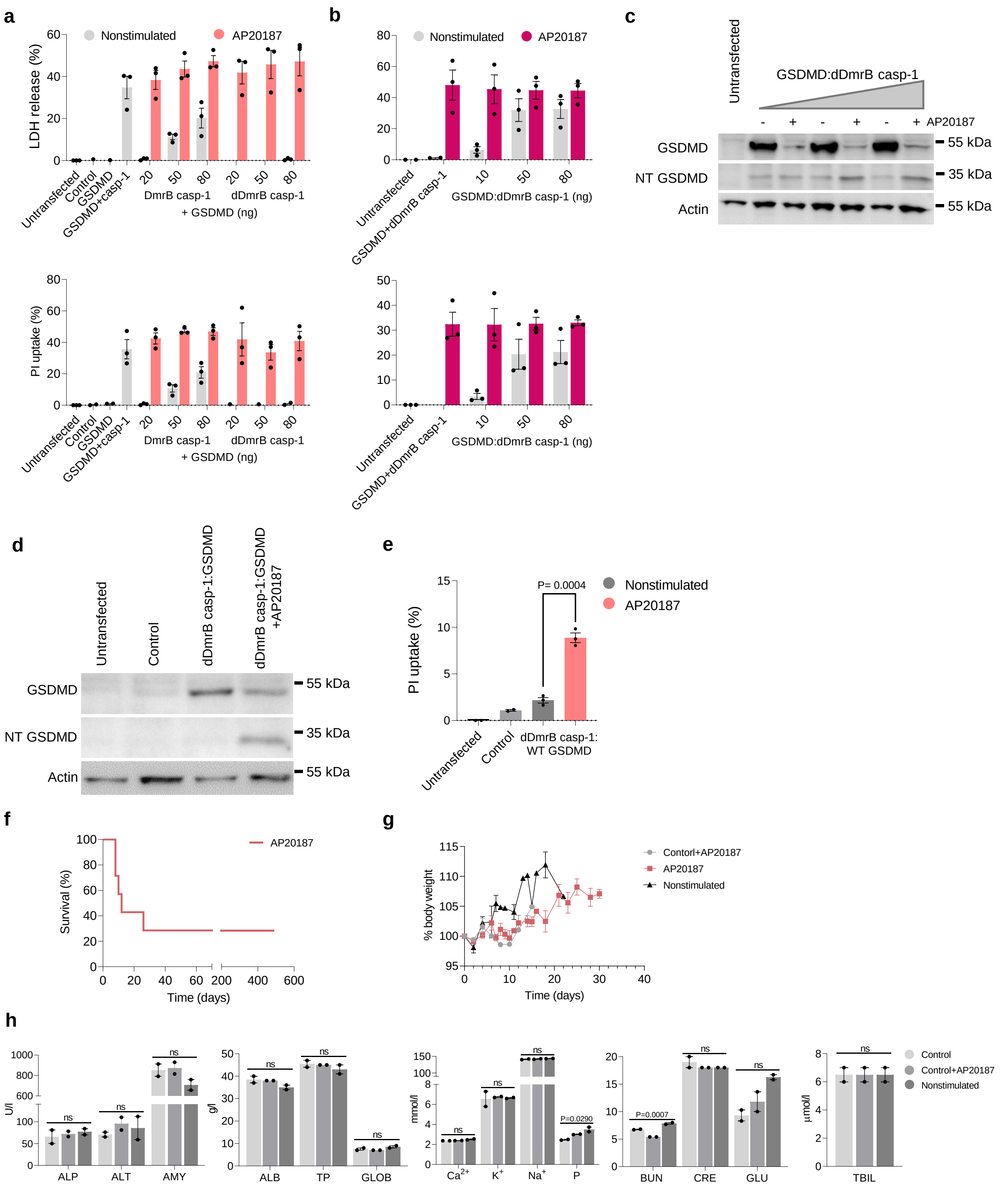

Supplementary Figure 5: Design of a dDmrB casp-1:GSDMD system. a Optimization of the dDmrB casp-1:GSDMD system. Casp-1ΔCARD was linked with one or two DmrB domains. Ligand-induced DmrB homodimerization and proteolytic activity of both casp-1ΔCARD constructs were tested on HEK293T cells and dimerization was initiated by the addition of 500 nM AP20187. **b** Validation of GSDMD:dDmrb casp-1 construct in HEK293T cells. **c** Western blot showing the full-length GSDMD and cleaved NT GSDMD in HEK293T cells after the GSDMD:dDmrB casp-1 transfection and AP20187-induced dimerization. **d** Western blot showing the full-length GSDMD and cleaved NT GSDMD in B16F10 cells after the dDmrB

casp-1:GSDMD transfection and AP20187-induced dimerization. **e** % of PI uptake in B16F10 cells transfected with dDmrB casp-1:GSDMD system. **f** Long-term survival of mice electroporated with dDmrB casp-1:GSDMD followed by i.t. AP20187 injection. **g** Normalized body weight of mice followed during the dDmrB casp-1:GSDMD experiment. **h** Biochemical evaluation of mouse sera after the treatment with the empty vector as a control, empty vector electroporation and AP20187 or electroporation of dDmrB casp-1:GSDMD. Abbreviations: ALP (alkaline phosphatase), ALT (alanine aminotransferase), AMY (amylase), ALB (albumin), TP (total protein), GLOB (globulin), Ca^{2+} (calcium), K^+ (potassium), Na^+ (sodium), P (phosphorus), BUN (urea nitrogen), CRE (creatinine), GLU (glucose), TBIL (total bilirubin). Data (**a, b, e**) is presented as mean ± SEM of three independent experiments. Representative blots (c, d) of three independent experiments are shown. The time (**f, g**) is defined as days after the first electroporation. Plots (**h**) show mean ± SEM (n=2) and a comparison between groups for each marker was done using one-way ANOVA. Source data are provided as a Source Data file.

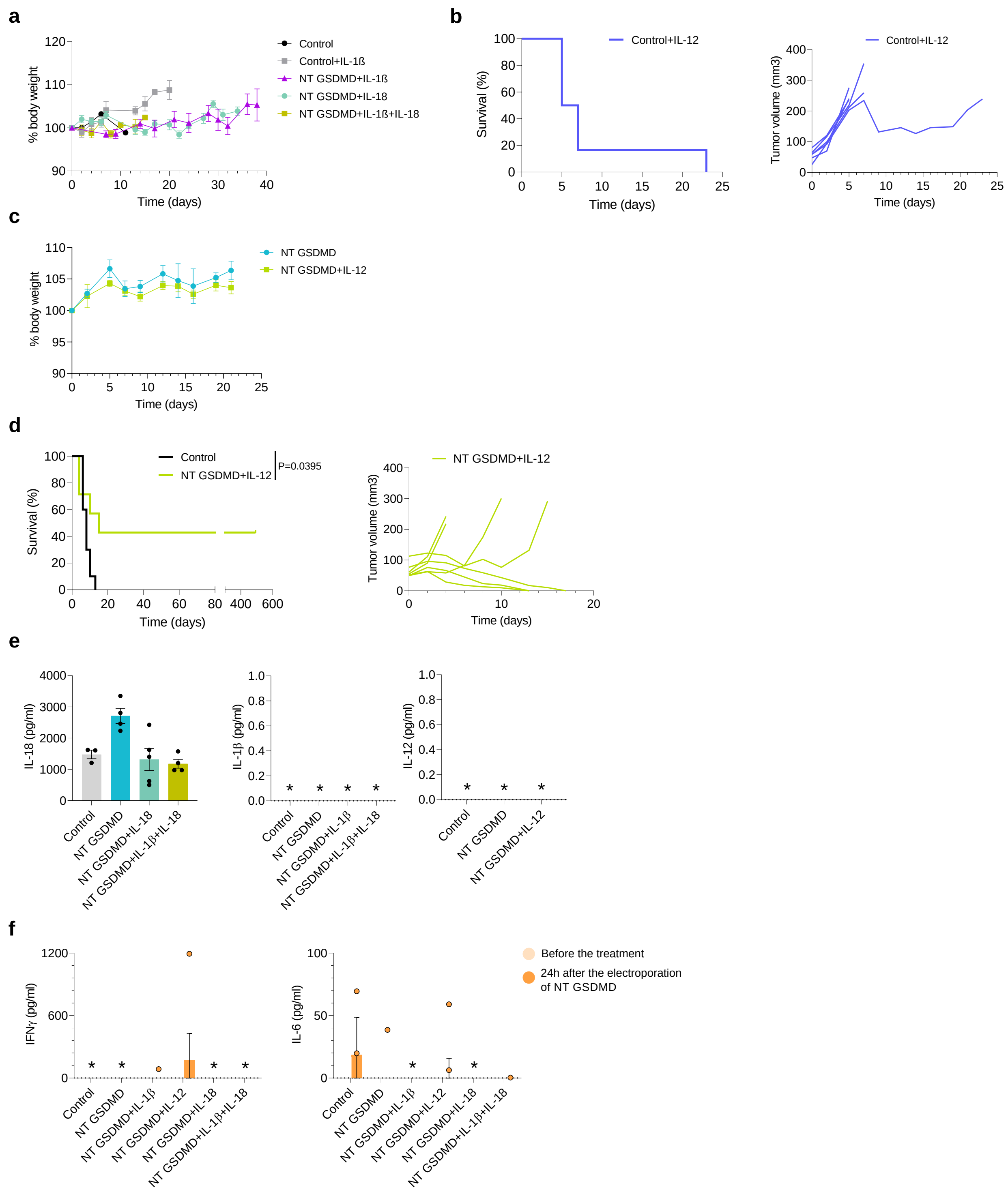

Supplementary Figure 6: Systemic effects of cytokine-armed pyroptosis. a Normalized body weight of mice followed during the deconstructed inflammasome experiment. **b** Survival and tumor growth following a single IL-12 electroporation. Tumor-bearing mice were electroporated with 20 µg plasmid coding for single-chain IL-12 fusion protein (n=6). Two days later empty plasmid (20 µg) electrotransfer followed. **c** Normalized body weight of mice followed during the IL-12-enriched pyroptosis experiment. **d** Long-term survival and tumor volume of mice treated with IL-12-enriched pyroptosis. Tumors were treated with a

plasmid coding for IL-12 and two days later with NT GSDMD (n=7) or an empty vector was electroporated as a control (n=10). The same control group as in Fig. 4c is shown. **e, f** Cytokine-armed pyroptosis does not affect cytokine levels in the sera. **e** IL-1β, IL-18, or IL-12 were measured in mice sera acquired 8 h post cytokine electroporation (day 0) in cytokine-treated groups and control and NT GSDMD group. **f** Sera from all experimental groups collected before the treatment initiation (day -4 before the first electroporation) and 24 h after the first NT GSDMD electroporation were analyzed for IFNγ and IL-6. Time (**a, b, c, d**) is shown as days succeeding the first treatment. Survival chart (**d**) was analyzed using a log-rank test. Plots (**e, f**) represent mean ± SEM of 3, 4, or 5 mice sera. * - values below the detection limit. Source data are provided as a Source Data file.

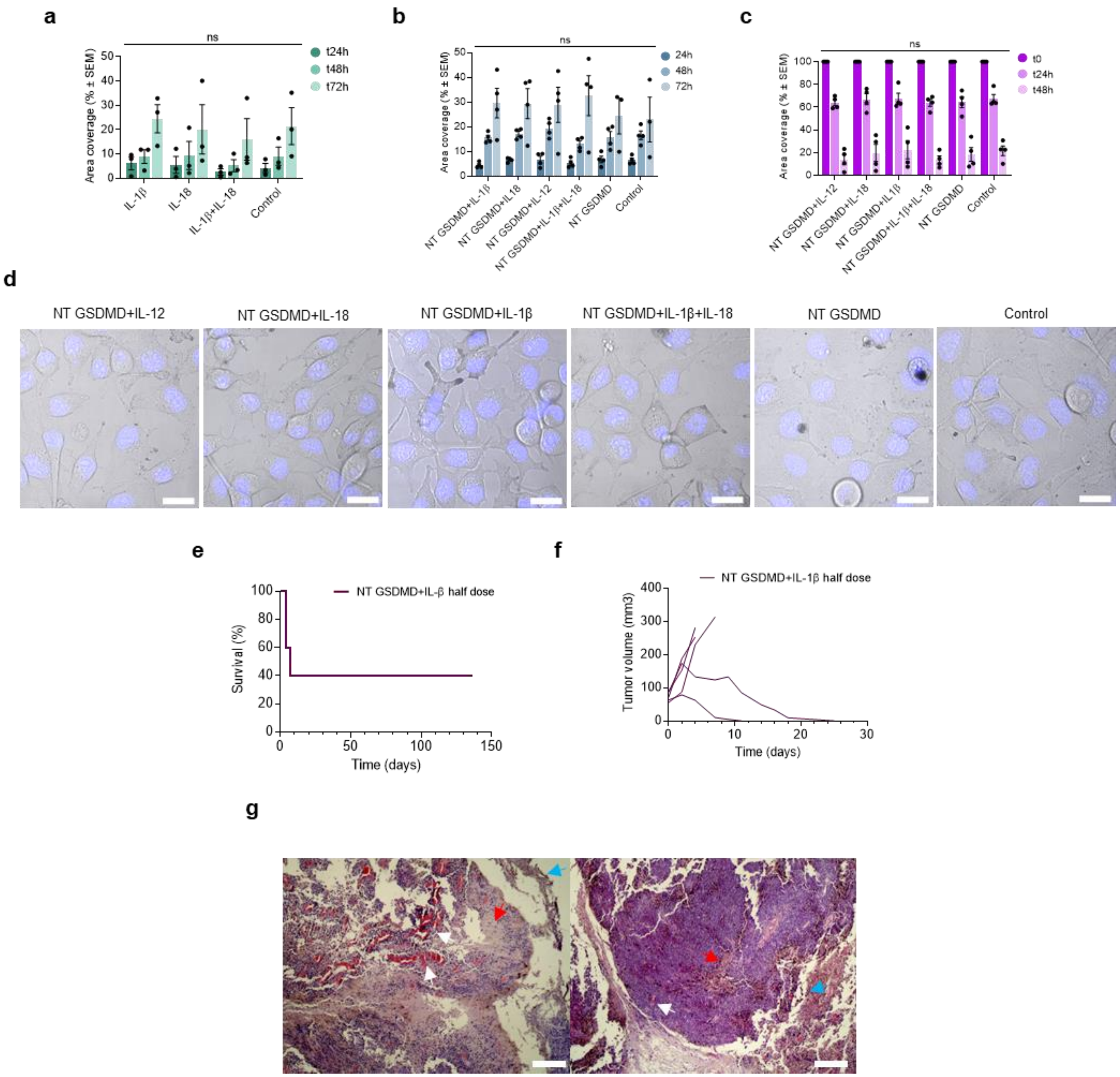

Supplementary Figure 7: Effect of different treatments on melanoma cells. a - d NT GSDMD and cytokine treatments do not alter the properties of cancer cells. **a** Proliferation rate of B16F10 cells electroporated with plasmids encoding IL-1β, IL-18, or IL-1β+IL-18. Proliferation rate (**b**), migration assay (**c**), and morphology (**d**) of primary cultures established from cytokine plasmid electroporated tumors. **d** Hoechst 33342 was used as a nuclear stain. Scale bars 20 µm. **e, f** Mice were treated according to a standard protocol, but instead of 20 µg of IL-1β plasmid only 10 µg was electoporated supplemented with 10 µg of empty vector two days before NT GSDMD. **g** HE-stained tumor tissue samples illustrating observed histological changes. White arrows indicate hyperemia, red arrows indicate loose connective tissue, and blue arrows indicate only partially visible and degenerated tumor capsule. Scale bars 50 µm. Left (NT GSDMD+IL-1β) and right (NT GSDMD+IL-18) images correspond to the same tumor samples as in Fig. 6a. Data in **a - c** are shown as the mean ± SEM of three or four independent experiments and statistical analysis was conducted utilizing two-way ANOVA with Turkey's multi-comparison test; ns - not significant. Time (**e, f**) is defined as days succeeding the first treatment. Source data are provided as a Source Data file.

a

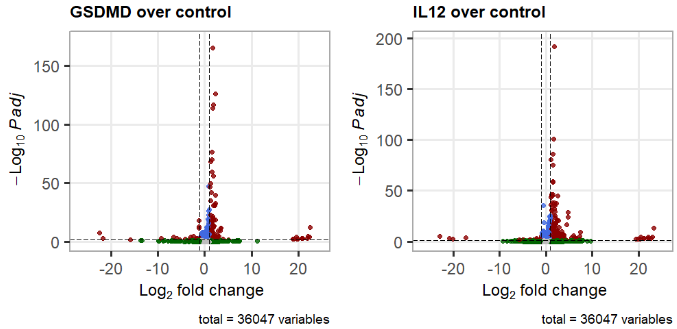

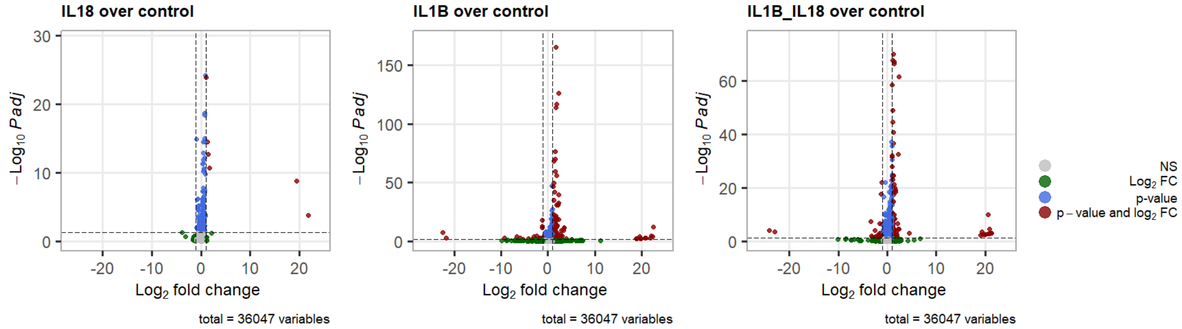

b

	p_{adj}	Log_2Fold Change			
Sample	< 0.05	> 0	> 1	< 0	< -1
GSDMD	304	134	38	170	21
IL1B_IL18	997	725	109	272	42
IL1B	783	580	188	203	28
IL18	380	319	6	61	0
IL12	1336	848	433	488	25

Supplementary Figure 8: Tumor transcriptomes after different treatments compared to control (empty vector treatment). **a** Volcano plots visualize the results of DESeq2 analyses of all samples. Thresholds for adjusted P and log_2FC were set to 0.05 and 1.0, respectively. In total 36047 variables (transcripts) were used for analyses. Tumor samples electroporated with an empty vector were used as a control. **b** Table shows the number of significantly differentially expressed genes with adjusted p value < 0.05 for all samples and out of those, number of upregulated genes with log_2FC above 0 and above 1, and number of downregulated genes with log_2FC below 0 and below -1.

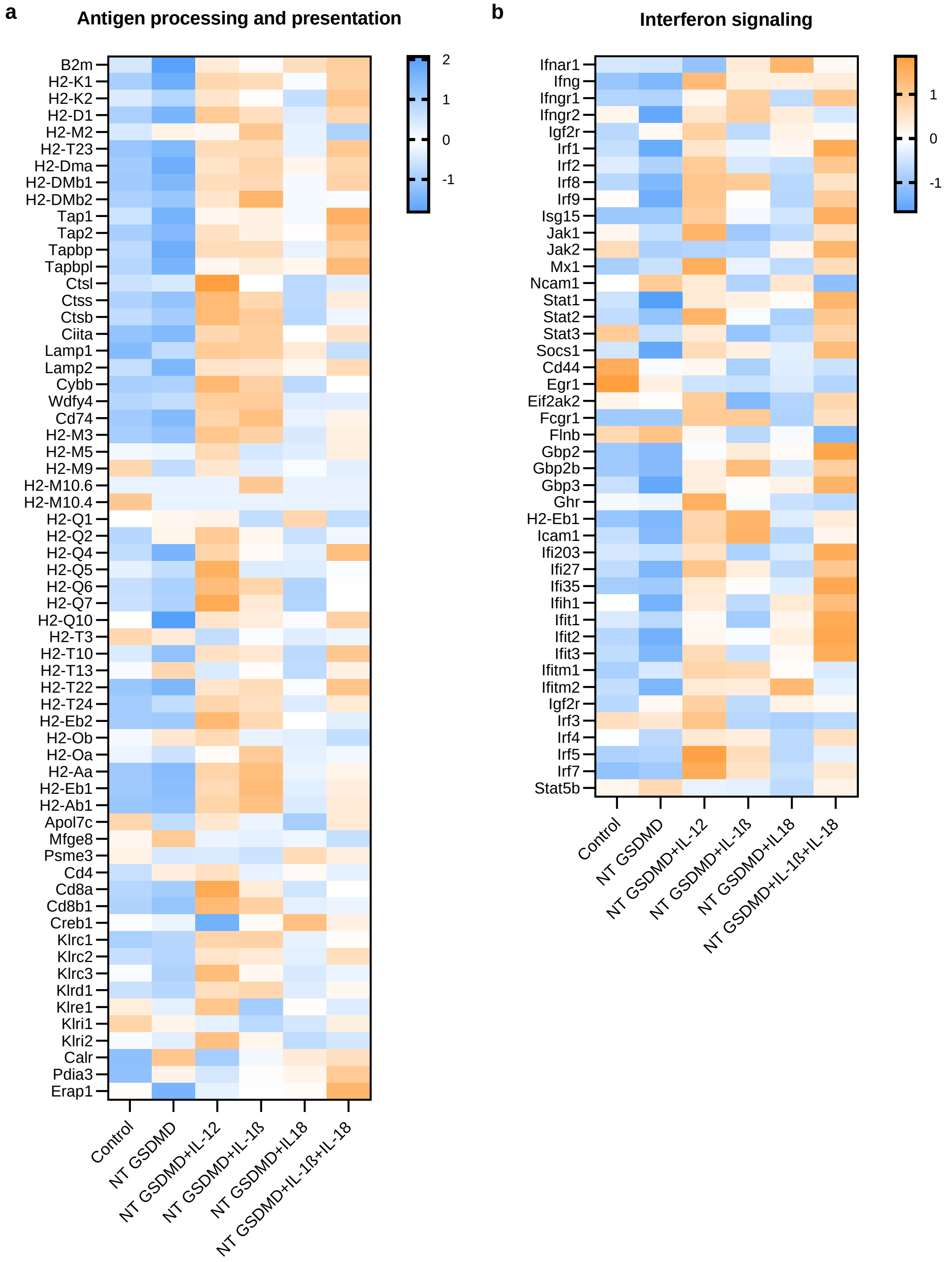

Supplementary Figure 9: NT GSDMD+IL-1β+IL-18 treatment does not lead to the downregulation of MHC I molecules and antigen presentation. a, b Differential gene expression. The heatmaps show genes involved in antigen processing and presentation (**a**) and interferon signaling (**b**). Z-scores are calculated over all samples, using DESeq2-normalized data. Source data are provided as a Source Data file.

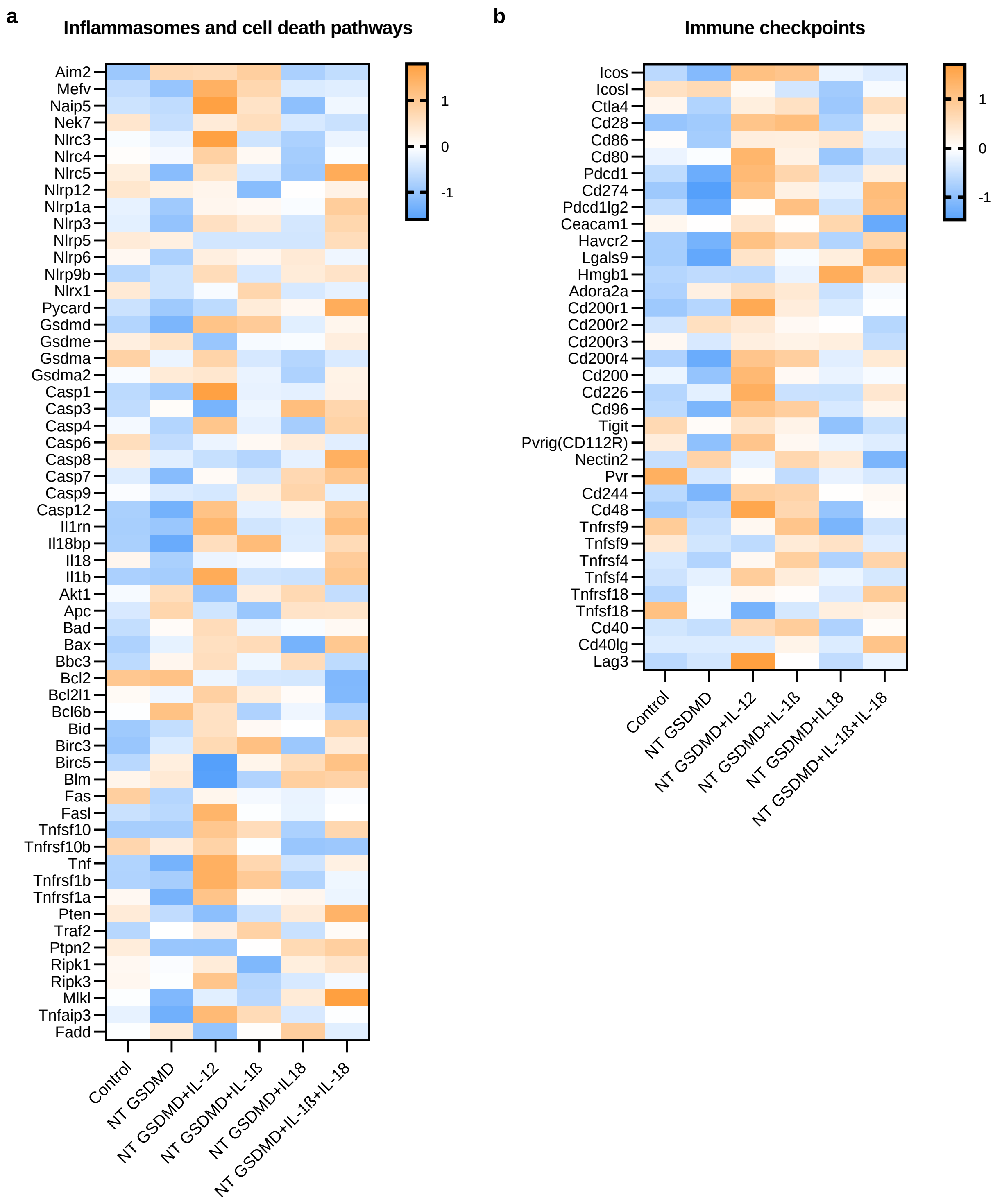

Supplementary Figure 10: NT GSDMD+IL-1β+IL-18 induces interferon signaling in treated tumors. **a, b** Differential gene expression of genes connected to inflammasome and cell death pathways (**a**), and immune checkpoints (**b**). Z-scores are calculated over all samples, using DESeq2-normalized data. Source data are provided as a Source Data file.

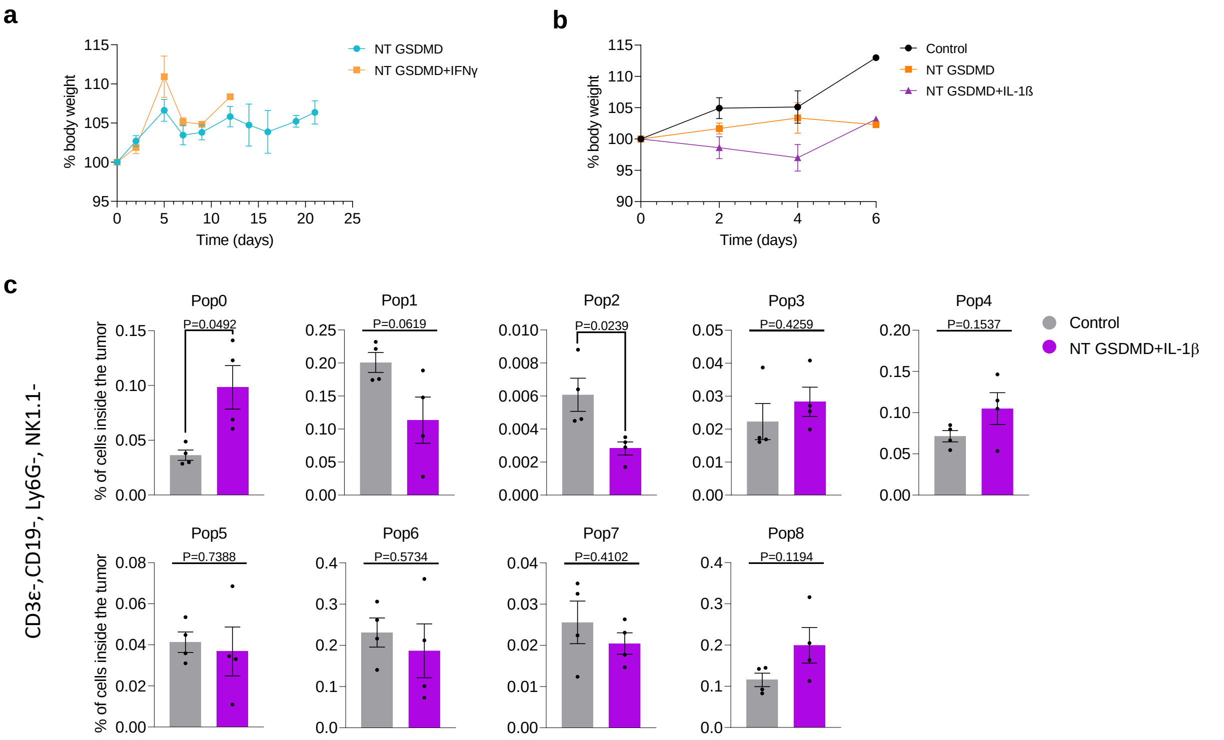

Pop0	CD103-	CD11cmed	MHCIImed	F4/80-	Ly6C-	CD11b-	CD24-
Pop1	CD103-	CD11c-	MHCII-	F4/80+	Ly6C-	CD11b+	CD24++
Pop2	CD103-	CD11c-	MHCII-	F4/80-	Ly6C-	CD11b-	CD24med
Pop3	CD103+	CD11c++	MHCII++	F4/80-	Ly6C-	CD11b-	CD24++
Pop4	CD103-	CD11cmed	MHCII++	F4/80+	Ly6C-	CD11b+	CD24med
Pop5	CD103-	CD11c-	MHCII-	F4/80+	Ly6C-	CD11b+	CD24-
Pop6	CD103-	CD11c-	MHCII-	F4/80med	Ly6C+	CD11b+	CD24med
Pop7	CD103-	CD11c++	MHCII++	F4/80-	Ly6C-	CD11b+	CD24med
Pop8	CD103-	CD11c-	MHCIImed	F4/80+	Ly6C+	CD11b+	CD24med

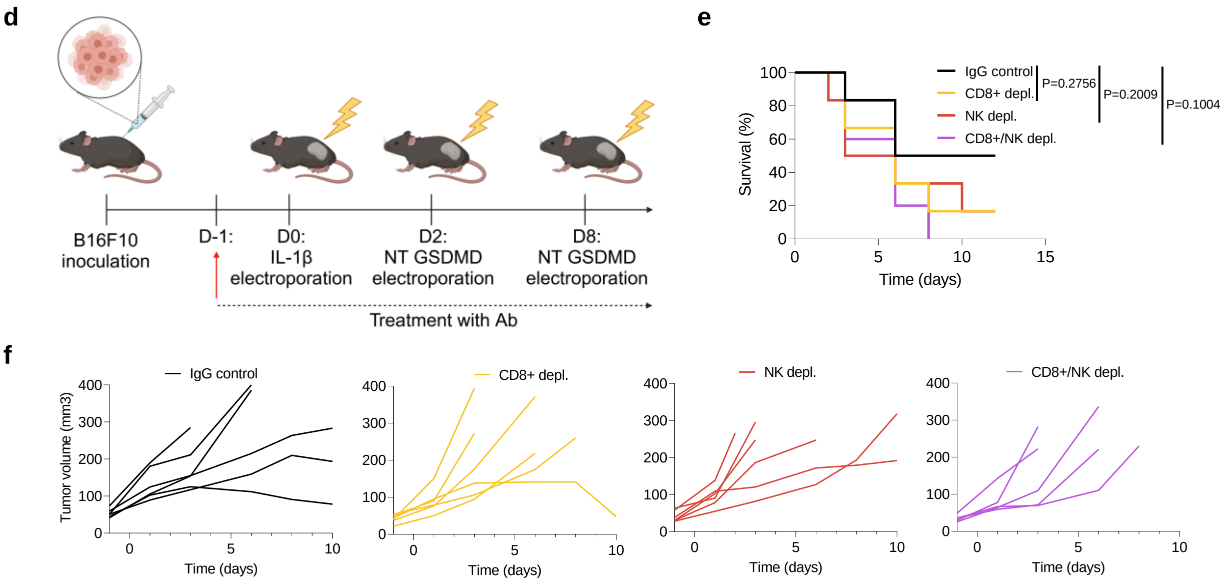

Supplementary Figure 11: Analysis of immune cell populations in melanoma tumors. Normalized body weight of mice followed during the NT GSDMD+IFNγ experiment (**a**) and experiment with immunocompromised mice (**b**). **c** Flow cytometry analysis of the cell populations inside the tumor. **d**

Timeline of depletion assay. Mice were depleted of NK1.1+ (n=6), CD8+ (n=6), and NK1.1+ together with CD8+ (n=5) cells by i.p. injection of appropriate antibodies or injected with control IgG (n=6), 1 day prior first electroporation, 1 day after, and repeated every 6th day until remission or euthanization. Survival data depicted as Kaplan-Meier curves (**e**) and tumor volume (**f**) indicating the impact of NK and/or CD8+ T cell depletion on NT GSDMD+IL-1β treatment. Time in days succeeding the first treatment is shown (**a, b, e, f**). Plots (**c**) are presented as mean ± SEM of four different tumors per group and were statistically evaluated with unpaired two-tailed t-test with Welch's correction in case of different variances. Survival plot (**e**) was analyzed using log-rank test. Source data are provided as a Source Data file. Supplementary Fig. 11d created in BioRender. Hafner Bratkovic, I. (2024) https://BioRender.com/b72f808.

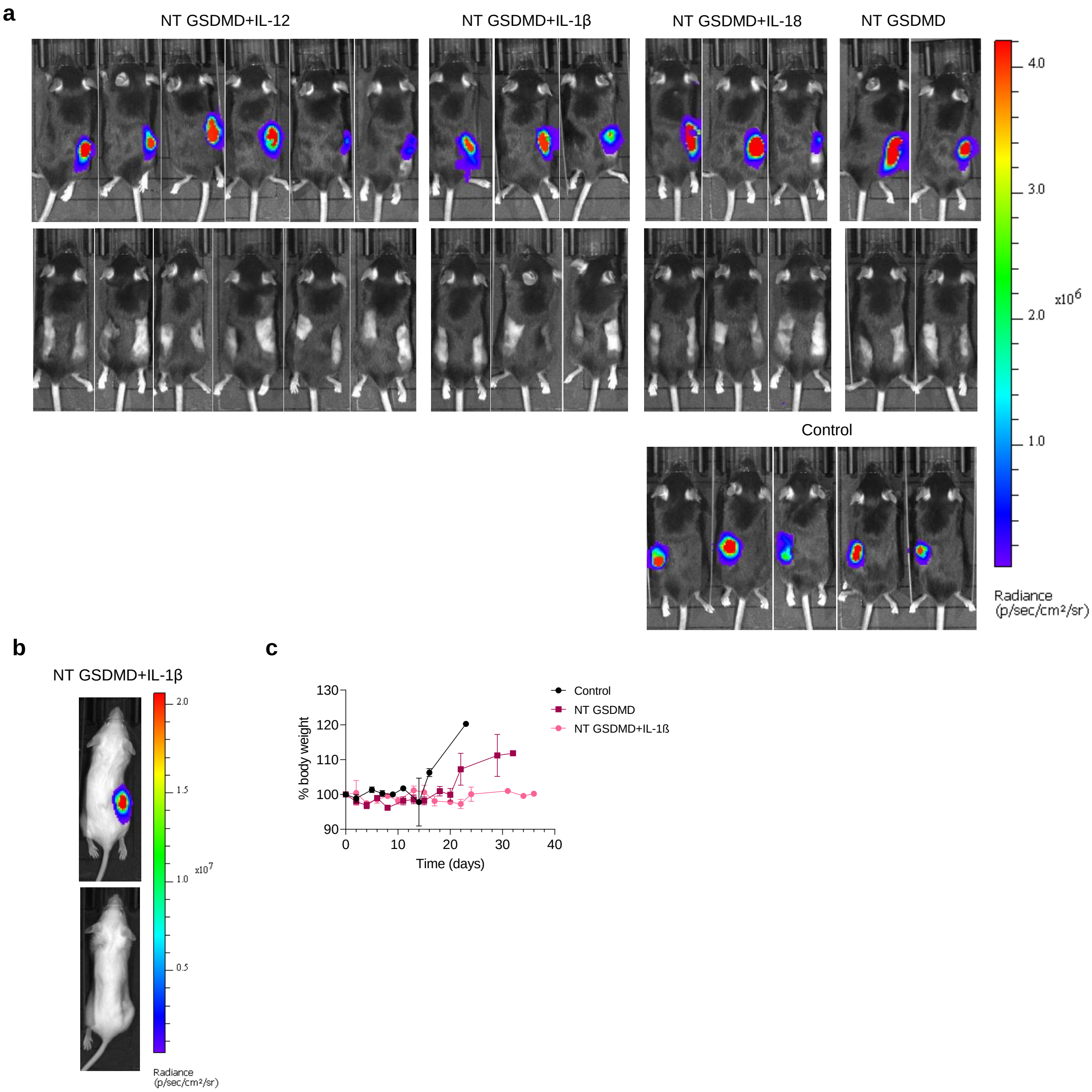

Supplementary Figure 12: In vivo bioluminescence imaging of B16F10 and 4T1 tumor model. a In vivo bioluminescence imaging of C57BL/6 mice bearing primary B16F10 melanoma tumors before the treatment (top) and mice in remission without primary or secondary tumors (middle) 10 days post rechallenge. Naïve control mice were injected with secondary tumors only (bottom) and imaged 10 days post-secondary tumor inoculation. **b** In vivo bioluminescence imaging of BALB/c mice bearing 4T1 breast cancer tumors before the treatment (top) and mouse in remission (bottom) following the NT GSDMD+IL-1β treatment. **c** Normalized body weight of mice followed during 4T1 tumor experiment. Time is presented as days following the first treatment. Source data are provided as a Source Data file.

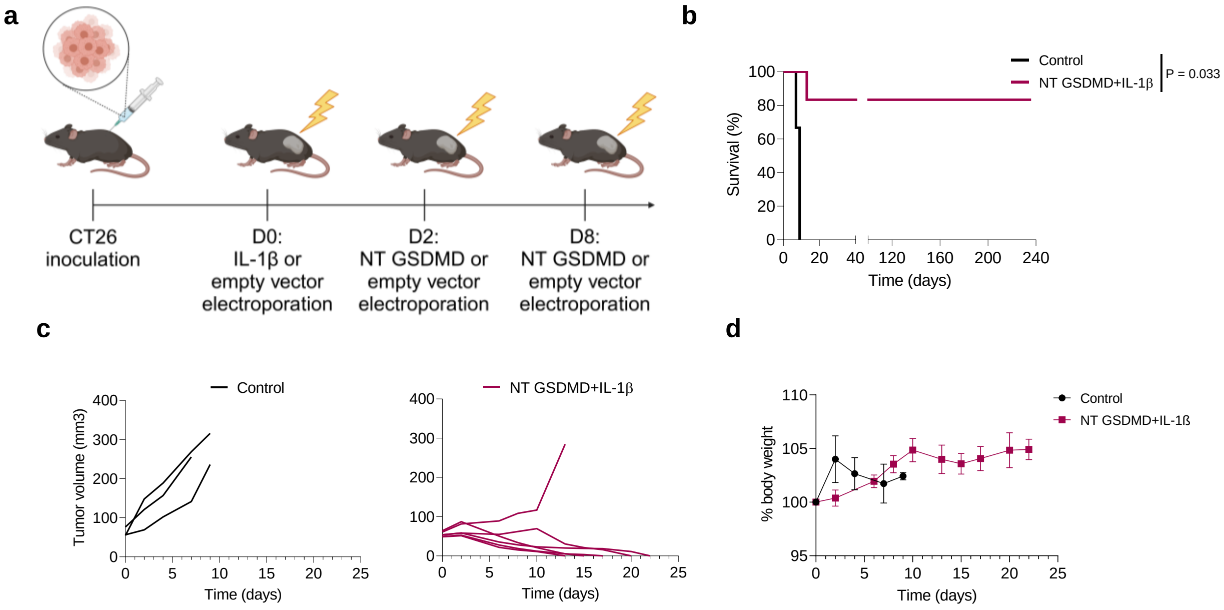

Supplementary Figure 13: Cytokine-armed pyroptosis for immunotherapy of hot tumors. a Timeline of CT26 tumor challenge experiment. CT26 tumor cells were s.c. engrafted into the C57BL/6J mice. When the tumors were palpable, IL-1β plasmid was electroporated followed by NT GSDMD two days later (n=6). Electroporation of an empty vector was used as a control (n=3). Survival (**b**), tumor volume (**c**), and normalized body weight charts (**d**) in which time is presented as days after the first treatment. Log-rank test was employed for the analysis of Kaplan-Meier curves (**b**). Source data are provided as a Source Data file. Supplementary Fig. 13a created in BioRender. Hafner Bratkovic, I. (2024) https://BioRender.com/f30n064.

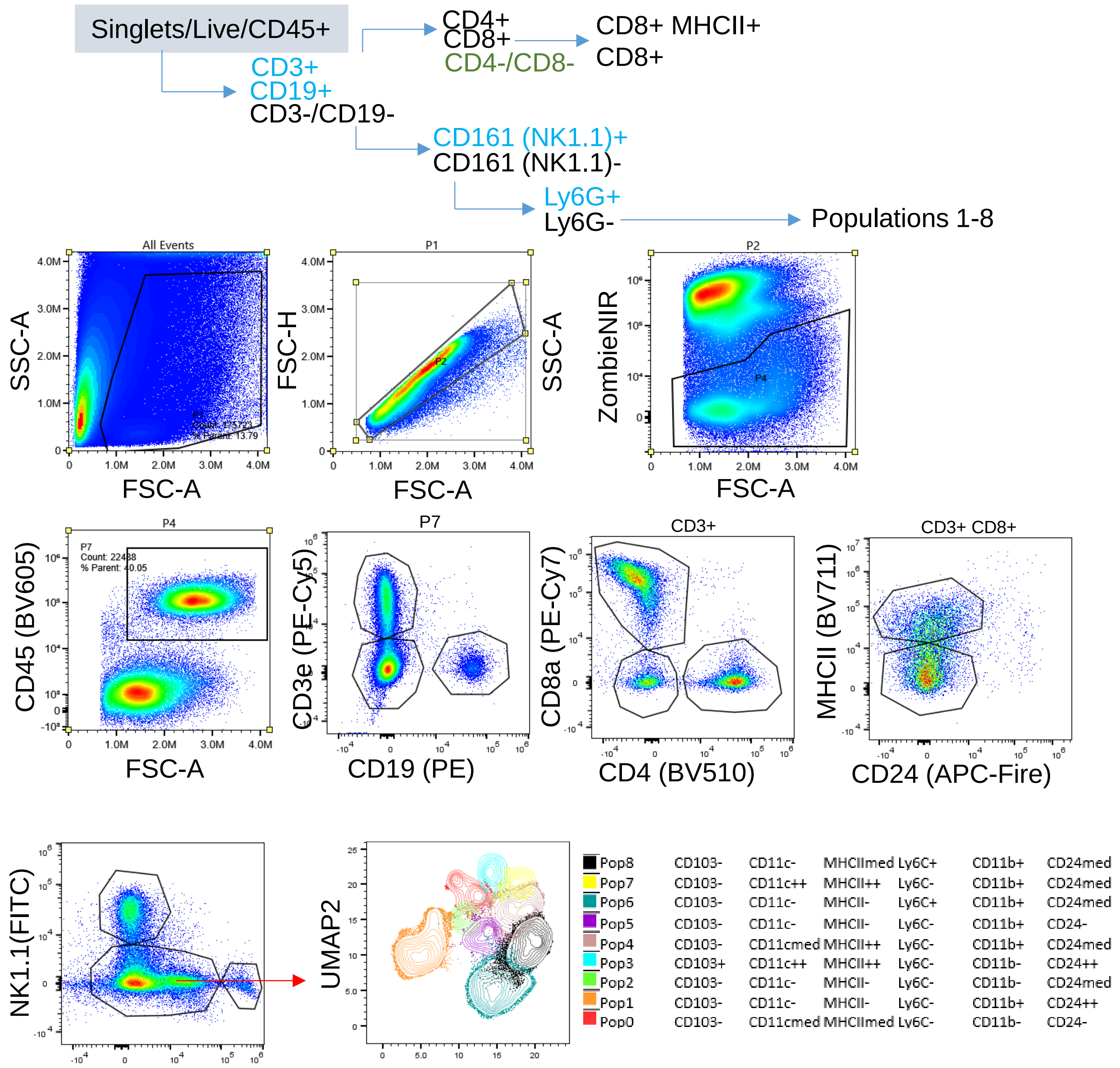

Supplementary Figure 14: Gating strategy for analysis of immune cell populations in B16F10 tumors. Gating strategy for Fig. 8e and Supplementary Fig. 11c.

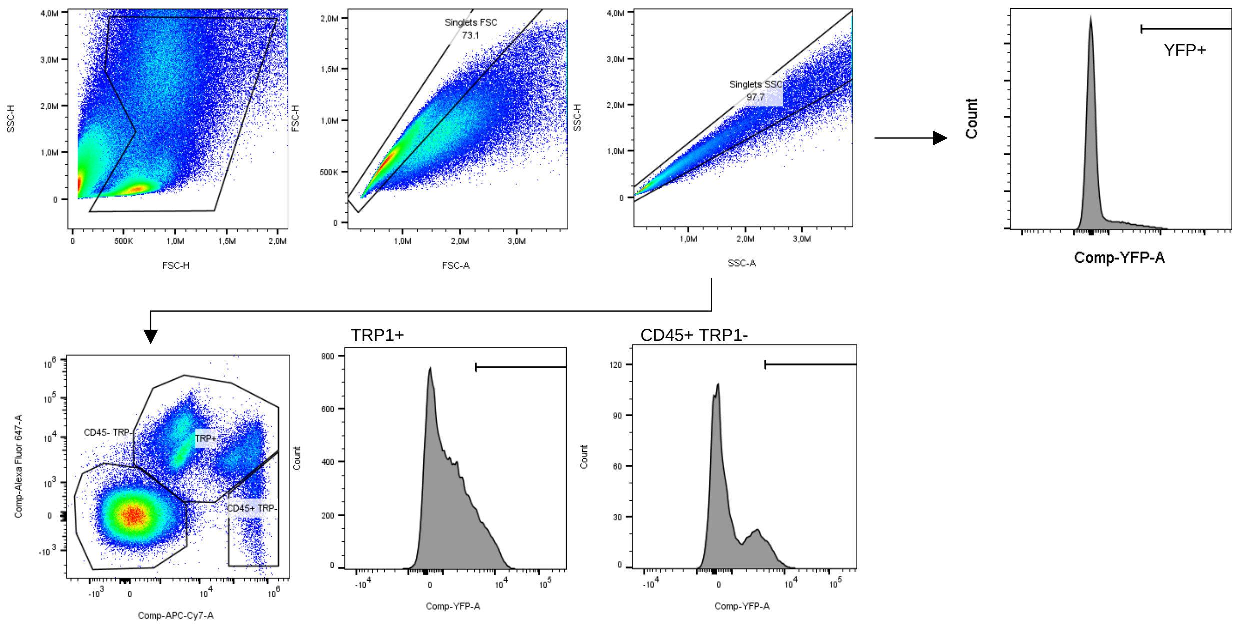

Supplementary Figure 15: Gating strategy for electroporation efficiency assay. Gating strategy for Supplementary Fig. 3b, c.

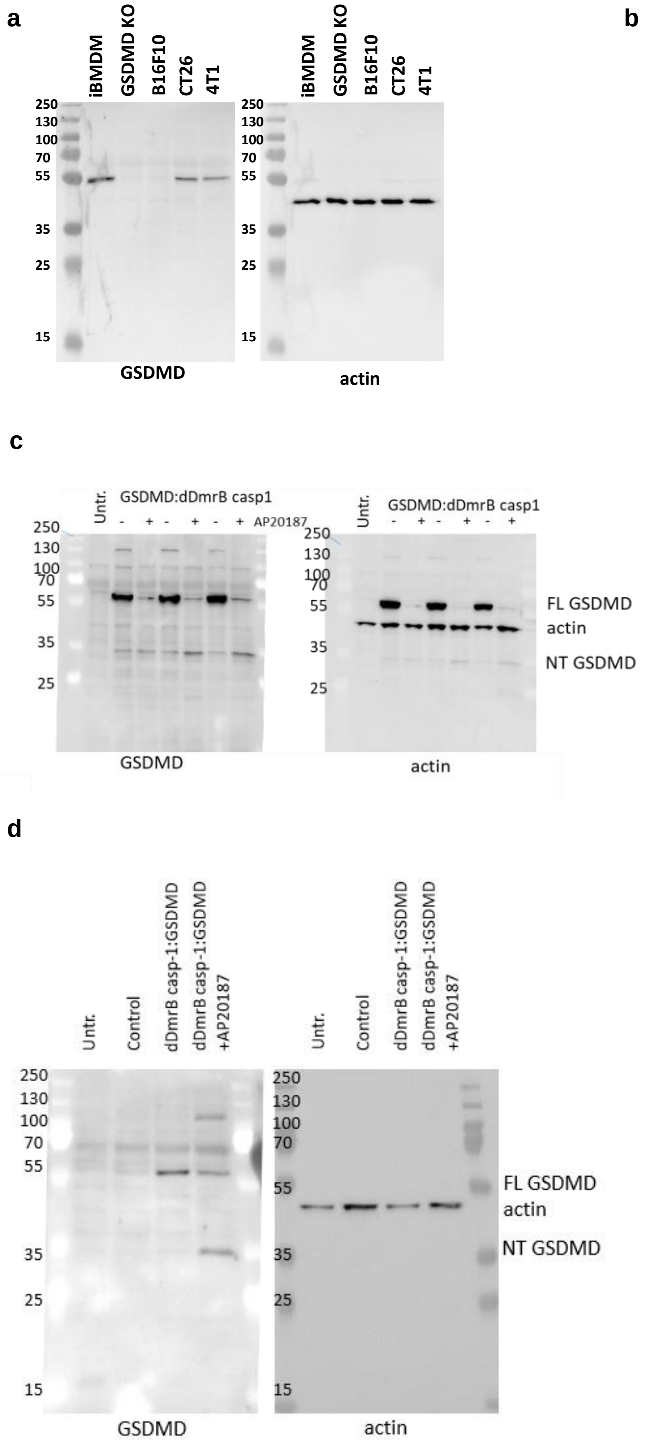

Supplementary Figure 16: Uncropped western blots. Uncropped western blots of Supplementary Fig. 1a (**a**) and 1b (**b**), Supplementary Fig. 5c (**c**), and Supplementary Fig. 5d (**d**).

Supplementary Table 1: Table of constructs

Name of the construct	Amino acid sequence
WT GSDMD	MPSAFEKVVKNVIKEVSGSRGDLIPVDSLRNSTSFRPYCLLNRKFSSSRFWKPRYS<br>CVNLSIKDILEPSAPEPEPECFGSFKVSDVVDGNIQGRVMLSGMGEGKISGGAAVS<br>DSSSASMNVCILRVTQKTWETMQHERHLQQPENKILQQLRSRGDDLFVVTEVLQT<br>KEEVQITEVHSQEGSGQFTLPGALCLKGEGKGHQSRKKMVTIPAGSILAFRVAQLLI<br>GSKWDILLVSDEKQRTFEPSSGDRKAVGQRHHGLNVLAALCSIGKQLSLLSDGIDE<br>EELIEAADFQGLYAEVKACSSELESLEMELRQQILVNIGKILQDQPSMEALEASLGQ<br>GLCSGGQVEPLDGPAGCILECLVLDSGELVPELAAPIFYLLGALAVLSETQQQLLAK<br>ALETTVLSKQLELVKHVLEQSTPWQEQSSVSLPTVLLGDCWDEKNPTWVLLEECG<br>LRLQVESPQVHWEPTSLIPTSALYASLFLLSSLGQKPC
NT GSDMD	MPSAFEKVVKNVIKEVSGSRGDLIPVDSLRNSTSFRPYCLLNRKFSSSRFWKPRYS<br>CVNLSIKDILEPSAPEPEPECFGSFKVSDVVDGNIQGRVMLSGMGEGKISGGAAVS<br>DSSSASMNVCILRVTQKTWETMQHERHLQQPENKILQQLRSRGDDLFVVTEVLQT<br>KEEVQITEVHSQEGSGQFTLPGALCLKGEGKGHQSRKKMVTIPAGSILAFRVAQLLI<br>GSKWDILLVSDEKQRTFEPSSGDRKAVGQRHHGLNVLAALCSIGKQLSLLSD
GSDMD I105N	MPSAFEKVVKNVIKEVSGSRGDLIPVDSLRNSTSFRPYCLLNRKFSSSRFWKPRYS<br>CVNLSIKDILEPSAPEPEPECFGSFKVSDVVDGNIQGRVMLSGMGEGKNSGGAAV<br>SDSSSASMNVCILRVTQKTWETMQHERHLQQPENKILQQLRSRGDDLFVVTEVLQ<br>TKEEVQITEVHSQEGSGQFTLPGALCLKGEGKGHQSRKKMVTIPAGSILAFRVAQL<br>LIGSKWDILLVSDEKQRTFEPSSGDRKAVGQRHHGLNVLAALCSIGKQLSLLSD
GSDMD TEV	MPSAFEKVVKNVIKEVSGSRGDLIPVDSLRNSTSFRPYCLLNRKFSSSRFWKPRYS<br>CVNLSIKDILEPSAPEPEPECFGSFKVSDVVDGNIQGRVMLSGMGEGKISGGAAVS<br>DSSSASMNVCILRVTQKTWETMQHERHLQQPENKILQQLRSRGDDLFVVTEVLQT<br>KEEVQITEVHSQEGSGQFTLPGALCLKGEGKGHQSRKKMVTIPAGSILAFRVAQLLI<br>GSKWDILLVSDEKQRTFEPSSGDRKAVGQRHHGLNVLAALCSIGKQENLYFQGID<br>EEELIEAADFQGLYAEVKACSSELESLEMELRQQILVNIGKILQDQPSMEALEASLG<br>QGLCSGGQVEPLDGPAGCILECLVLDSGELVPELAAPIFYLLGALAVLSETQQQLLA<br>KALETTVLSKQLELVKHVLEQSTPWQEQSSVSLPTVLLGDCWDEKNPTWVLLEEC<br>GLRLQVESPQVHWEPTSLIPTSALYASLFLLSSLGQKPC
DmrB-casp1	MGVQVETISPGDGRTFPKRGQTCVVHYTGMLEDGKKVDSSRDRNKPFKFMLGKQ<br>EVIRGWEEGVAQMSVGQRAKLTISPDYAYGATGHPGIIPPHATLVFDVELLKLEGG<br>SGGSAPSAETFVATEDSKGGHPSSSETKEEQNKEDGTFPGLTGTLKFCPLEKAQK<br>LWKENPSEIYPIMNTTTRTRLALIICNTEFQHLSPRVGAQVDLREMKLLLEDLGYTV<br>KVKENLTALEMVKEVKEFAACPEHKTSDSTFLVFMSHGIQEGICGTTYSNEVSDILK<br>VDTIFQMMNTLKCPSLKDKPKVIIIQACRGEKQGVVLLKDSVRDSEEDFLTDAIFED<br>DGIKKAHIEKDFIAFCSSTPDNVSWRHPVRGSLFIESLIKHMKEYAWSCDLEDIFRK<br>VRFSFEQPEFRLQMPTADRVTLTKRFYLFPGDPVP
dDmrB-casp1	MGVQVETISPGDGRTFPKRGQTCVVHYTGMLEDGKKVDSSRDRNKPFKFMLGKQ<br>EVIRGWEEGVAQMSVGQRAKLTISPDYAYGATGHPGIIPPHATLVFDVELLKLETR<br>GVQVETISPGDGRTFPKRGQTCVVHYTGMLEDGKKVDSSRDRNKPFKFMLGKQE<br>VIRGWEEGVAQMSVGQRAKLTISPDYAYGATGHPGIIPPHATLVFDVELLKLEGGS<br>GGSAPSAETFVATEDSKGGHPSSSETKEEQNKEDGTFPGLTGTLKFCPLEKAQKL<br>WKENPSEIYPIMNTTTRTRLALIICNTEFQHLSPRVGAQVDLREMKLLLEDLGYTVK<br>VKENLTALEMVKEVKEFAACPEHKTSDSTFLVFMSHGIQEGICGTTYSNEVSDILKV<br>DTIFQMMNTLKCPSLKDKPKVIIIQACRGEKQGVVLLKDSVRDSEEDFLTDAIFEDD<br>GIKKAHIEKDFIAFCSSTPDNVSWRHPVRGSLFIESLIKHMKEYAWSCDLEDIFRKV<br>RFSFEQPEFRLQMPTADRVTLTKRFYLFPGDPVP
dDmrB-<br>casp1:T2A:GSDMD	MGVQVETISPGDGRTFPKRGQTCVVHYTGMLEDGKKVDSSRDRNKPFKFMLGKQ<br>EVIRGWEEGVAQMSVGQRAKLTISPDYAYGATGHPGIIPPHATLVFDVELLKLETR<br>GVQVETISPGDGRTFPKRGQTCVVHYTGMLEDGKKVDSSRDRNKPFKFMLGKQE<br>VIRGWEEGVAQMSVGQRAKLTISPDYAYGATGHPGIIPPHATLVFDVELLKLEGGS<br>GGSAPSAETFVATEDSKGGHPSSSETKEEQNKEDGTFPGLTGTLKFCPLEKAQKL<br>WKENPSEIYPIMNTTTRTRLALIICNTEFQHLSPRVGAQVDLREMKLLLEDLGYTVK<br>VKENLTALEMVKEVKEFAACPEHKTSDSTFLVFMSHGIQEGICGTTYSNEVSDILKV<br>DTIFQMMNTLKCPSLKDKPKVIIIQACRGEKQGVVLLKDSVRDSEEDFLTDAIFEDD<br>GIKKAHIEKDFIAFCSSTPDNVSWRHPVRGSLFIESLIKHMKEYAWSCDLEDIFRKV<br>RFSFEQPEFRLQMPTADRVTLTKRFYLFPGDPVPGSGEGRGSLLTCGDVEENPGP<br>PSAFEKVVKNVIKEVSGSRGDLIPVDSLRNSTSFRPYCLLNRKFSSSRFWKPRYSC<br>VNLSIKDILEPSAPEPEPECFGSFKVSDVVDGNIQGRVMLSGMGEGKISGGAAVSD

	SSSASMNVCILRVTQKTWETMQHERHLQQPENKILQQLRSRGDDLFVVTEVLQTK<br>EEVQITEVHSQEGSGQFTLPGALCLKGEGKGHQSRKKMVTIPAGSILAFRVAQLLI<br>GSKWDILLVSDEKQRTFEPSSGDRKAVGQRHHGLNVLAALCSIGKQLSLLSDGIDE<br>EELIEAADFQGLYAEVKACSSELESLEMELRQQILVNIGKILQDQPSMEALEASLGQ<br>GLCSGGQVEPLDGPAGCILECLVLDSGELVPELAAPIFYLLGALAVLSETQQQLLAK<br>ALETTVLSKQLELVKHVLEQSTPWQEQSSVSLPTVLLGDCWDEKNPTWVLLEECG<br>LRLQVESPQVHWEPTSLIPTSALYASLFLLSSLGQKPC
GSDMD:T2A:dDmrb-casp1	MPSAFEKVVKNVIKEVSGSRGDLIPVDSLRNSTSFRPYCLLNRKFSSSRFWKPRYS<br>CVNLSIKDILEPSAPEPEPECFGSFKVSDVVDGNIQGRVMLSGMGEGKISGGAAVS<br>DSSSASMNVCILRVTQKTWETMQHERHLQQPENKILQQLRSRGDDLFVVTEVLQT<br>KEEVQITEVHSQEGSGQFTLPGALCLKGEGKGHQSRKKMVTIPAGSILAFRVAQLLI<br>GSKWDILLVSDEKQRTFEPSSGDRKAVGQRHHGLNVLAALCSIGKQLSLLSDGIDE<br>EELIEAADFQGLYAEVKACSSELESLEMELRQQILVNIGKILQDQPSMEALEASLGQ<br>GLCSGGQVEPLDGPAGCILECLVLDSGELVPELAAPIFYLLGALAVLSETQQQLLAK<br>ALETTVLSKQLELVKHVLEQSTPWQEQSSVSLPTVLLGDCWDEKNPTWVLLEECG<br>LRLQVESPQVHWEPTSLIPTSALYASLFLLSSLGQKPCGSGEGRGSLLTCGDVEEN<br>PGPGVQVETISPGDGRTFPKRGQTCVVHYTGMLEDGKKVDSSRDRNKPFKFMLG<br>KQEVIRGWEEGVAQMSVGQRAKLTISPDYAYGATGHPGIIPPHATLVFDVELLKLE<br>TRGVQVETISPGDGRTFPKRGQTCVVHYTGMLEDGKKVDSSRDRNKPFKFMLGK<br>QEVIRGWEEGVAQMSVGQRAKLTISPDYAYGATGHPGIIPPHATLVFDVELLKLEG<br>GSGGSAPSAETFVATEDSKGGHPSSSETKEEQNKEDGTFPGLTGTLKFCPLEKAQ<br>KLWKENPSEIYPIMNTTTRTRLALIICNTEFQHLSPRVGAQVDLREMKLLLEDLGYT<br>VKVKENLTALEMVKEVKEFAACPEHKTSDSTFLVFMSHGIQEGICGTTYSNEVSDIL<br>KVDTIFQMMNTLKCPSLKDKPKVIIIQACRGEKQGVVLLKDSVRDSEEDFLTDAIFE<br>DDGIKKAHIEKDFIAFCSSTPDNVSWRHPVRGSLFIESLIKHMKEYAWSCDLEDIFR<br>KVRFSFEQPEFRLQMPTADRVTLTKRFYLFPGDPVP
FKBP cTEV	MGVQVETISPGDGRTFPKRGQTCVVHYTGMLEDGKKFDSSRDRNKPFKFMLGKQ<br>EVIRGWEEGVAQMSVGQRAKLTISPDYAYGATGHPGIIPPHATLVFDVELLKLEGS<br>GSKSMSSMVSDTSCTFPSSDGIFWKHWIQTKDGQCGSPLVSTRDGFIVGIHSASN<br>FTNTNNYFTSVPKNFMELLTNQEAQQWVSGWRLNADSVLWGGHKVFMSKPEEP<br>FQPVKEATQLMSELVYSQ
FRB nTEV	MILWHEMWHEGLEEASRLYFGERNVKGMFEVLEPLHAMMERGPQTLKETSFNQA<br>YGRDLMEAQEWCRKYMKSGNVKDLLQAWDLYYHVFRRISKGSGSGESLFKGPR<br>DYNPISSTICHLTNESDGHTTSLYGIGFGPFIITNKHLFRRNNGTLLVQSLHGVFKVK<br>NTTTLQQHLIDGRDMIIIRMPKDFPPFPQKLKFREPQREERICLVTTNFQT
IL-1β HA-tag	MVPIRQLHYRLRDEQQKSLVLSDPYELKALHLNGQNINQQVIFSMSFVQGEPSNDK<br>IPVALGLKGKNLYLSCVMKDGTPTLQLESVDPKQYPKKKMEKRFVFNKIEVKSKVE<br>FESAEFPNWYISTSQAEHKPVFLGNNSGQDIIDFTMESVSSGSGYPYDVPDYA
IL-18 HA-tag	MNFGRLHCTTAVIRNINDQVLFVDKRQPVFEDMTDIDQSASEPQTRLIIYMYKDSEV<br>RGLAVTLSVKDSKMSTLSCKNKIISFEEMDPPENIDDIQSDLIFFQKRVPGHNKMEF<br>ESSLYEGHFLACQKEDDAFKLILKKKDENGDKSVMFTLTNLHQSGGGGSYPYDVP<br>DYA
IL-12 p40p30	MCPQKLTISWFAIVLLVSPLMAMWELEKDVYVVEVDWTPDAPGETVNLTCDTPEE<br>DDITWTSDQRHGVIGSGKTLTITVKEFLDAGQYTCHKGGETLSHSHLLLHKKENGI<br>WSTEILKNFKNKTFLKCEAPNYSGRFTCSWLVQRNMDLKFNIKSSSSSPDSRAVTC<br>GMASLSAEKVTLDQRDYEKYSVSCQEDVTCPTAEETLPIELALEARQQNKYENYST<br>SFFIRDIIKPDPPKNLQMKPLKNSQVEVSWEYPDSWSTPHSYFSLKFFVRIQRKKEK<br>MKETEEGCNQKGAFLVEKTSTEVQCKGGNVCVQAQDRYYNSSCSKWACVPCRV<br>RSGGGGSGGGGSGGGGSRVIPVSGPARCLSQSRNLLKTTDDMVKTAREKLKHYS<br>CTAEDIDHEDITRDQTSTLKTCLPLELHKNESCLATRETSSTTRGSCLPPQKTSLMM<br>TLCLGSIYEDLKMYQTEFQAINAALQNHNHQQIILDKGMLVAIDELMQSLNHNGETL<br>RQKPPVGEADPYRVKMKLCILLHAFSTRVVTINRVMGYLSSA
IFNγ	MNATHCILALQLFLMAVSGCYCHGTVIESLESLNNYFNSSGIDVEEKSLFLDIWRNW<br>QKDGDMKILQSQIISFYLRLFEVLKDNQAISNNISVIESHLITTFFSNSKAKKDAFMSI<br>AKFEVNNPQVQRQAFNELIRVVHQLLPESSLRKRKRSRC
FLAG-tag YFP	MDYKDDDDKEFCRYPVSKGEELFTGVVPILVELDGDVNGHKFSVSGEGEGDATY<br>GKLTLKFICTTGKLPVPWPTLVTTFGYGLQCFARYPDHMKQHDFFKSAMPEGYVQ<br>ERTIFFKDDGNYKTRAEVKFEGDTLVNRIELKGIDFKEDGNILGHKLEYNYNSHNVYI<br>MADKQKNGIKVNFKIRHNIEDGSVQLADHYQQNTPIGDGPVLLPDNHYLSYQSALS<br>KDPNEKRDHMVLLEFVTAAGITLGMDELYK

Supplementary Table 2: Table of oligonucleotides

Name	Nucleotide sequence
GSDMD I105_F	ggagaagggaaaaattctggtggggct
GSDMD I105_R	agccccaccagaatttttcccttctcc
GSDMD TEV_F	catcggaaagcaggagaatctctacttccaggggattgatgagg
GSDMD TEV_R	cctcatcaatcccctggaagtagagattctcctgctttccgatg
GSDMD WT:T2A:dDmrB casp-1_F	gtctaggccagaaaccttgtggttctggagagggcagaggcagtctgctgacatgcggtgacgtggaa gagaatcccggccctggcgtccaagtcgaaaccattagtc
GSDMD WT:T2A:dDmrB casp-1_R	cgccagtgtgatggatatctgcagaattccctatggtaccggatcccccggg
dDmrB casp-1:T2A:GSDMD WT_F	tcccgggggatccggtaccaggttctggagagggcagaggcagtctgctgacatgcggtgacgtgga agagaatcccggccctccatcggcctttgagaaagtggtc
dDmrB casp-1:T2A:GSDMD WT _R	gtgtgatggatatctgcagaattccctaacaaggtttctggcctagacttgac
mINFgamma_F	cggatccgccaccatgaacgctacacactgcatct
mINFgamma_R	ggaattcctcagcagcgactccttttcc
mIL-18-HA_F	cttggtaccgagctcggatccgccaccatgaactttggccgacttcac
mIL-18-HA_R	gtgatggatatctgcagaattcttaggcgtagtcaggcacgtcgtaaggataagagcctccaccccccac tttgatgtaagtt
mIL-12 p40p35_F	caagcttggtaccgagctcggatccgccaccatgtgtcctcagaagctaac
mIL-12 p40p35_R	cgccagtgtgatggatatctgcagaattcctcaggcggagctcagatagc
