# Supplementary material for: Cytokine-armed pyroptosis induces antitumor immunity against diverse types of tumors
Source: Nat Commun. 2024 Dec 30;15:10801. doi: 10.1038/s41467-024-55083-3 (PMC11686184; doi:10.1038/s41467-024-55083-3)
Supplement: Supplementary file 2 — Reporting Summary [file 41467_2024_55083_MOESM2_ESM.pdf]

nature portfolio

Corresponding author(s): Iva Hafner Bratkovič

Last updated by author(s): Nov 12, 2024

Reporting Summary

Nature Portfolio wishes to improve the reproducibility of the work that we publish. This form provides structure for consistency and transparency in reporting. For further information on Nature Portfolio policies, see our Editorial Policies and the Editorial Policy Checklist.

Statistics

For all statistical analyses, confirm that the following items are present in the figure legend, table legend, main text, or Methods section.

n/a	Confirmed	
☐	☒	The exact sample size (*n*) for each experimental group/condition, given as a discrete number and unit of measurement
☐	☒	A statement on whether measurements were taken from distinct samples or whether the same sample was measured repeatedly
☐	☒	The statistical test(s) used AND whether they are one- or two-sided<br>*Only common tests should be described solely by name; describe more complex techniques in the Methods section.*
☐	☒	A description of all covariates tested
☐	☒	A description of any assumptions or corrections, such as tests of normality and adjustment for multiple comparisons
☐	☒	A full description of the statistical parameters including central tendency (e.g. means) or other basic estimates (e.g. regression coefficient) AND variation (e.g. standard deviation) or associated estimates of uncertainty (e.g. confidence intervals)
☐	☒	For null hypothesis testing, the test statistic (e.g. *F*, *t*, *r*) with confidence intervals, effect sizes, degrees of freedom and *P* value noted<br>*Give P values as exact values whenever suitable.*
☒	☐	For Bayesian analysis, information on the choice of priors and Markov chain Monte Carlo settings
☒	☐	For hierarchical and complex designs, identification of the appropriate level for tests and full reporting of outcomes
☒	☐	Estimates of effect sizes (e.g. Cohen's *d*, Pearson's *r*), indicating how they were calculated

Our web collection on statistics for biologists contains articles on many of the points above.

Software and code

Policy information about availability of computer code

Data collection	Bulk RNA sequencing data was collected on Illumina® NovaSeqTM. In vivo bioluminescence data collection was performed using IVIS Lumina Series III (Perkin Elmer). LA-ICP-MS data was collected with a laser ablation system (193 nm ArF* excimer; Analyte G2 Teledyne Photon Machines Inc., Bozeman, MT) coupled to a quadrupole ICP-MS instrument (Agilent 7900x, Agilent Technologies, Santa Clara, CA). For Western blot G-box (Syngene) and Genesnap 7.09 software were used. Imaging of primary cell cultures was done using TCS SP5 confocal microscope (Leica). Cell proliferation and migration assay was performed with CellInsight CX7 (Thermo Scientific) as well as cell visualization. In vivo bioluminescence imaging was monitored by IVIS Lumina Series III (Perkin Elmer). PI uptake and LDH cytotoxicity assay were measured with multiplate reader SinergyMx (BioTek) and Gen 5.1.10 software (BioTek). Mice sera was analyzed with VetSCcan Chemistry Analyzer (Abaxis).
Data analysis	Bulk RNA sequencing data was analyzed with tools within the RNAlysis software. QC was performed using FastQCheck and CutAdapt 105 was utilized for paired-end adapter trimming. Transcripts were identified using Kallisto 106 tool for pseudoalignment of trimmed samples to the mouse genome, utilizing pre-produced index and gtf files, downloaded from Kallisto transcriptome indices data source (version June 22 2019; kallisto 0.45.1, Ensembl v96 transcriptomes; https://github.com/pachterlab/kallisto-transcriptome-indices/releases; accessed Sep 29 2023). For differential expression and relevant statistical analysis, we used RStudio (RStudio 2023.09.1+494) R-package DESeq2. Data were normalized with an in-built function (median-of-ratios method), the threshold of significance was set to adjusted P-value ≤ 0.1. If not stated otherwise, ggplot2 package was used to draw plots. Heatmaps were drawn with GraphPad Prism 8, with Z-scores, calculated from DESeq normalized data. For enrichment analysis we used significantly upregulated genes of each sample (adjusted P-value <0.1 and log2FC > 0.5) as an input to the online-available WEB-based Gene Set Analysis toolkit (WebGestalt) and performed Over-Representation Analysis (ORA), choosing functional database gene ontology (non-redundant biological process) and the reference set (mouse) genome. In vivo bioluminescence data was analyzed using Living Image 4.7.3 (PerkinElmer). LA-ICP-MS data processing and image analysis were performed using the software package HDIP (Teledyne Photon Machines Inc., Bozeman, MT). Cell proliferation and migration assay data was analyzed using ImageJ software. Statistical analysis of data was performed using GraphPad Prism 8 software (GraphPad software, Inc.). Graphs were

generated with GraphPad Prism 8 software (GraphPad software, Inc.).

For manuscripts utilizing custom algorithms or software that are central to the research but not yet described in published literature, software must be made available to editors and reviewers. We strongly encourage code deposition in a community repository (e.g. GitHub). See the Nature Portfolio guidelines for submitting code & software for further information.

Data

Policy information about availability of data

All manuscripts must include a data availability statement. This statement should provide the following information, where applicable:

- Accession codes, unique identifiers, or web links for publicly available datasets
- A description of any restrictions on data availability
- For clinical datasets or third party data, please ensure that the statement adheres to our policy

All data generated and supporting the findings in this study are available within the paper. Raw RNA sequencing data and corresponding processed data files have been deposited in the Gene Expression Omnibus under the accession code GSE261286.

Research involving human participants, their data, or biological material

Policy information about studies with human participants or human data. See also policy information about sex, gender (identity/presentation), and sexual orientation and race, ethnicity and racism.

Reporting on sex and gender	*Use the terms sex (biological attribute) and gender (shaped by social and cultural circumstances) carefully in order to avoid confusing both terms. Indicate if findings apply to only one sex or gender; describe whether sex and gender were considered in study design; whether sex and/or gender was determined based on self-reporting or assigned and methods used.*<br>*Provide in the source data disaggregated sex and gender data, where this information has been collected, and if consent has been obtained for sharing of individual-level data; provide overall numbers in this Reporting Summary. Please state if this information has not been collected.*<br>*Report sex- and gender-based analyses where performed, justify reasons for lack of sex- and gender-based analysis.*
Reporting on race, ethnicity, or other socially relevant groupings	*Please specify the socially constructed or socially relevant categorization variable(s) used in your manuscript and explain why they were used. Please note that such variables should not be used as proxies for other socially constructed/relevant variables (for example, race or ethnicity should not be used as a proxy for socioeconomic status).*<br>*Provide clear definitions of the relevant terms used, how they were provided (by the participants/respondents, the researchers, or third parties), and the method(s) used to classify people into the different categories (e.g. self-report, census or administrative data, social media data, etc.)*<br>*Please provide details about how you controlled for confounding variables in your analyses.*
Population characteristics	*Describe the covariate-relevant population characteristics of the human research participants (e.g. age, genotypic information, past and current diagnosis and treatment categories). If you filled out the behavioural & social sciences study design questions and have nothing to add here, write "See above."*
Recruitment	*Describe how participants were recruited. Outline any potential self-selection bias or other biases that may be present and how these are likely to impact results.*
Ethics oversight	*Identify the organization(s) that approved the study protocol.*

Note that full information on the approval of the study protocol must also be provided in the manuscript.

Field-specific reporting

Please select the one below that is the best fit for your research. If you are not sure, read the appropriate sections before making your selection.

[x] Life sciences [ ] Behavioural & social sciences [ ] Ecological, evolutionary & environmental sciences

For a reference copy of the document with all sections, see nature.com/documents/nr-reporting-summary-flat.pdf

Life sciences study design

All studies must disclose on these points even when the disclosure is negative.

Sample size	Biomath (InVivoStat) and G power 3.1 (Heinrich-Heine-Universität Düsseldorf, Germany) software were used to predetermine sample size for mice experiments.
Data exclusions	No data was excluded from the study.
Replication	Biological replicates are noted in the figure legends or methods. Mice studies were tipically performed twice and each study was designed to use the minimal number of mice. Three independent experiments were performed for in vitro stuides. For bulk RNA sequencing, collected RNA from the animals of the same group was pulled together and sent for sequencing in triplicates.
Randomization	Animals were randomized before the treatment ranked by tumor volume to alternate treatment groups. Mice with no detectable tumor or

mice with tumors exceeding humane endpoints were not included in the study.

Blinding: The researchers were not blinded during the experiments or data collection and analysis, except for histological, flow cytometry, and LA-ICP-MS analysis of tumor samples.

Reporting for specific materials, systems and methods

We require information from authors about some types of materials, experimental systems and methods used in many studies. Here, indicate whether each material, system or method listed is relevant to your study. If you are not sure if a list item applies to your research, read the appropriate section before selecting a response.

Materials & experimental systems

n/a	Involved in the study	
☐	☒	Antibodies
☐	☒	Eukaryotic cell lines
☒	☐	Palaeontology and archaeology
☐	☒	Animals and other organisms
☒	☐	Clinical data
☒	☐	Dual use research of concern
☒	☐	Plants

Methods

n/a	Involved in the study	
☒	☐	ChIP-seq
☐	☒	Flow cytometry
☒	☐	MRI-based neuroimaging

Antibodies

Antibodies used: PD-1 blocking antibodies (anti-mouse CD279, clone RMP1-14, P362, Leinco Technologies), rat IgG2a (clone 1-1, product no. I-1177, Leinco Technologies), CTLA-4 blocking antibodies (anti-mouse CTLA-4, clone 9D9, C2855, Leinco Technologies), mouse IgG2b isotype control (clone MCP-11, product no. I-119, Leinco Technologies), anti-mouse CD8b.2 monoclonal antibody (clone 53-5.8, C2832, Leinco Technologies), anti-mouse NK1.1 monoclonal antibody (clone PK136, N123, Leinco Technologies) or mouse IgG2a (I-118, Leinco Technologies) were used for mice studies. For Western blot primary anti-GSDMD (Abcam, ab209845), anti-caspase-1 p20 antibody (Casper-1, Adipogen, AG-20B-0042-C100), anti-ASC antibody (AL177, Adipogen, AG-25B-0006) and anti-NLRP3 antibodies (Cryo2, Adipogen, AG-20B-0014-C100), and anti-β-Actin (Cell Signaling Technology, 8H10D10) α/β-Tubulin Antibody Cell Signaling Technology (#2148) antibodies were used, and secondary HRP-conjugated anti-rabbit (Jackson ImmunoResearch, 111-035-003) and HRP-conjugated anti-mouse (Jackson ImmunoResearch, 115-035-003). Anti-mouse CD8a (53-6.7)-146Nd (Standard BioTools, 3146003B) were utilized for tissue staining for LA-ICP-MS. For flow cytometry the following antibodies were used: CD103 (clone 2E7)-BV421 (Biolegend 121421, 1:100), CD24 (clone M1/69)-APC-Fire750 (Biolegend, 101839, 1:100), CD4(clone RM4-5)-BV510 (Biolegend 100553, 1:200), CD11b (clone M1/70)-AF700 (eBioscience 56-0112, 1:200), CD8a(clone 53-6.7)-PE-Cy7 (Biolegend 100721, 1:200), F4/80(BM8)-PE-Dazzle594 (Biolegend 123145, 1:100), MHCII (cloneM5/114.15.2)-BV711, Biolegend 107643, 1:200), CD19(clone eBio1D3)-PE (eBioscience 12-0193-82, 1:200), CD161 (NK1.1) (clone S17016D)-FITC (Biolegend 156508, 1:100), CD11c (clone N418)-BV570 (Biolegend, 117331, 1:200), CD3 (clone 145-2C11)-PE-Cy5, Invitrogen 15-0031-82, 1:200), CD64 (clone X54-5/7.1)-PerCP-Cy5.5 (Biolegend 139307, 1:100), Ly-6C (HK1.4)-APC (Milteny 130-102-341, 1:50), Ly-6G (1A8)-BV785 (Biolegend 127645, 1:200), CD45 (30-F11)-BV605 (Biolegend 103139,1:200), CD11c (clone N418)-BV605 (Biolegend, 117334, 1:100), CD3ε (clone 145-2C11)-BV711 (Biolegend, 100349, 1:200), CD11b (clone M1/70)-BV750 (Biolegend, 101267, 1:200), CD45 (clone 30-F11)- APC-Cy7 (Biolegend, 103115, 1:500), TRP1- Alexa Fluor 647 (abcam, ab270105, 1:100).

Validation: All antibodies were validated by manufacturer as indicated on their website.

Eukaryotic cell lines

Policy information about cell lines and Sex and Gender in Research

Cell line source(s): HEK293T cells were obtained from the American Type Culture Collection (ATCC, CRL-3216). Bioware® Brite Cell Line B16F10 Red-FLuc cells were ordered from PerkinElmer (cat.no. BW124734). Murine BALB/c colorectal carcinoma cell line CT26 and murine BALB/c breast cancer cell line 4T1 cells were a kind gift from Prof. M. Čemažar (Institute of Oncology, Ljubljana). CT26 Red-FLuc and 4T1 Red-FLuc were generated by lentivirus transduction. The generation of immortalized bone marrow-derived macrophages (iBMDM) from wild-type and GSDMD-KO mice was previously described (Evavold, C. L., Hafner-Bratkovic et al. Control of gasdermin D oligomerization and pyroptosis by the Ragulator-Rag-mTORC1 pathway. Cell 184, 4495-4511 e4419, doi:10.1016/j.cell.2021.06.028 (2021))

Authentication: No specific authentication method other than evaluation by morphology was used.

Mycoplasma contamination: All cell lines were confirmed mycoplasma negative.

Commonly misidentified lines (See ICLAC register): No commonly misidentified cell lines were used.

Animals and other research organisms

Policy information about studies involving animals; ARRIVE guidelines recommended for reporting animal research, and Sex and Gender in Research

Laboratory animals	8-12 weeks old male and female C57BL/6J OlaHSd and B-NDG mice from the animal facility of National Institute of Chemistry Slovenia and 8-12 weeks old female BALB/c OlaHsd purchased from Envigo were maintained in 12-12 hour dark-light cycle at approximately 40-60% relative humidity with ambient temperature (22°C). All animals used in the study were healthy and accompanied by a health certificate from the animal vendor. Health/microbiological status was confirmed by FELASA recommended Mouse Vivum immunocompetent panel (QM Diagnostics).
Wild animals	Study did not involve wild animals.
Reporting on sex	Experiments using C57BL/6J OlaHsd and B-NDG mice were performed in female and male mice. Study using BALB/c OlaHsd mice was done on female mice.
Field-collected samples	Study did not involve field-collected samples.
Ethics oversight	All animal experiments were performed according to the 3R principles and directives of the EU 2010/63 and were approved by the Administration of the Republic of Slovenia for Food Safety, Veterinary, and Plant Protection of the Ministry of Agriculture, Forestry, and Foods, Republic of Slovenia (Permit Number U34401-9/2020/9, U34401-24/2023/4 and U34401-18/2023/8). Mice were humanely euthanized when the length, width, or height of the tumor exceeded 12 mm (a defined humane endpoint) or when the tumor showed signs of ulceration.

Note that full information on the approval of the study protocol must also be provided in the manuscript.

Plants

Seed stocks	*Report on the source of all seed stocks or other plant material used. If applicable, state the seed stock centre and catalogue number. If plant specimens were collected from the field, describe the collection location, date and sampling procedures.*
Novel plant genotypes	*Describe the methods by which all novel plant genotypes were produced. This includes those generated by transgenic approaches, gene editing, chemical/radiation-based mutagenesis and hybridization. For transgenic lines, describe the transformation method, the number of independent lines analyzed and the generation upon which experiments were performed. For gene-edited lines, describe the editor used, the endogenous sequence targeted for editing, the targeting guide RNA sequence (if applicable) and how the editor was applied.*
Authentication	*Describe any authentication procedures for each seed stock used or novel genotype generated. Describe any experiments used to assess the effect of a mutation and, where applicable, how potential secondary effects (e.g. second site T-DNA insertions, mosiacism, off-target gene editing) were examined.*

Flow Cytometry

Plots

Confirm that:

- [x] The axis labels state the marker and fluorochrome used (e.g. CD4-FITC).
- [x] The axis scales are clearly visible. Include numbers along axes only for bottom left plot of group (a 'group' is an analysis of identical markers).
- [x] All plots are contour plots with outliers or pseudocolor plots.
- [x] A numerical value for number of cells or percentage (with statistics) is provided.

Methodology

Sample preparation	Analysis of immune cell populations in tumors using flow cytometry: Tumor tissue was harvested 14 or 15 days after the B16F10 inoculation (4 or 5 days after the first NT GSDMD treatment, respectively), cut into smaller pieces and gentleMACS Dissociator (Miltenyi Biotec) was used for the preparation of a single-cell suspension according to the manufacturer's protocol. Cells were passed through 70 µm nylon strainers (VWR) and 0.5×106 of tumor cells were resuspended in FACS buffer (PBS supplemented with 10% FBS). For Live/Dead staining cells were incubated on ice for 10 min in 100 µl PBS containing ZombieNIR dye (dilution 1: 2000, Biolegend, 423106)). After removal of the dye, cells were resuspended in 50 µl FACS buffer containing anti-mouse CD16/CD32 (Fcγ III/II receptor) (1:25, BD Pharmingen). 10 min later, an antibody cocktail was added (50 µl) and cells were incubated on ice for at least 30 min. The antibody cocktail was prepared in FACS buffer containing True Stain Monocyte blocker (1:20, 426103, BioLegend) including the following antibodies: CD103 (clone 2E7)-BV421 (Biolegend 121421, 1:100), CD24 (clone M1/69)-APC-Fire750 (Biolegend, 101839, 1:100), CD4(clone RM4-5)-BV510 (Biolegend 100553, 1:200), CD11b (clone M1/70)-AF700 (eBioscience 56-0112, 1:200), CD8a(clone 53-6.7)-PE-Cy7 (Biolegend 100721, 1:200), F4/80(BM8)-PE-Dazzle594 (Biolegend 123145, 1:100), MHCII (cloneM5/114.15.2)-BV711, Biolegend 107643, 1:200), CD19(clone eBio1D3)-PE (eBioscience 12-0193-82, 1:200), CD161 (NK1.1) (clone S17016D)-FITC (Biolegend 156508, 1:100), CD11c (clone N418)-BV570 (Biolegend, 117331, 1:200), CD3 (clone 145-2C11)-PE-Cy5, Invitrogen 15-0031-82, 1:200), CD64 (clone X54-5/7.1)-PerCP-Cy5.5 (Biolegend 139307, 1:100), Ly-6C (HK1.4)-APC (Milteny 130-102-341, 1:50), Ly-6G (1A8)-

BV785 (Biolegend 127645, 1:200), CD45 (30-F11)-BV605 (Biolegend 103139,1:200). As reference controls, an unstained sample and for every marker, a single-stained reference control (spleen cells) was acquired.
Electroporation efficiency by flow cytometry:
Established B16F10 tumors were electroporated with 20 µg of plasmid coding for a yellow fluorescent protein (YFP) and tumors were collected 48 h later and the single cell suspension was prepared as stated above. 2×106 of tumor cells were resuspended in 50 µl of FACS buffer supplemented with 2 µl Mouse TrueSatin FcX (BioLegend) and incubated for 10 min at room temperature. Afterwards, 50 µl of antibody cocktail containing CD11c (clone N418)-BV605 (Biolegend, 117334, 1:100), CD3ε (clone 145-2C11)-BV711 (Biolegend, 100349, 1:200), CD11b (clone M1/70)-BV750 (Biolegend, 101267, 1:200), CD45 (clone 30-F11)- APC-Cy7 (Biolegend, 103115, 1:500), TRP1- Alexa Fluor 647 (abcam, ab270105, 1:100) in FACS buffer with 5 µl True Stain Monocyte blocker (BioLegend) was added and samples were incubated for at least 30 min (but no longer than 2 h) on ice. Before analysis cells were washed with FACS buffer and fluorescence was measured on an Aurora spectral flow cytometer (Cytek Biosciences). All antibodies were titrated individually according to standard practice before being used in the panel.
As reference controls, an unstained sample and, for every color, a single-stain reference control were acquired (for CD11c, CD1b, CD3, CD45 spleen cells 105 and for TRP1 B16F10 cells 105 were used). For YFP, B16 cells electroporated with a plasmid carrying YFP were used. All reference controls underwent the same protocol as fully stained samples, including washes.

Instrument

Aurora spectral flow cytometer (Cytek Bioscience)

Software

SpectroFlo v3.1.0 software (Cytek Bioscience)
FlowJo v. 10.10.0 software (BD Biosciences)
UMAP dimensionality reduction (version 4.1.1) method
FlowSOM (version 4.1.0)120 clustering approach

Cell population abundance

No sorting was used in the study.

Gating strategy

Analysis of immune cell populations in tumors using flow cytometry:
After the removal of doublets and dead cells, manual gates were imposed on different populations of CD45 positive cells, such as CD19-positive cells, CD3ε-positive cells. The CD45+ CD3ε- CD19- population was further separated into NK1.1 positive cells and Ly6G positive cells. The CD3-, CD19-, Ly6G-, and NK1.1- negative cell population was further analyzed using the UMAP dimensionality reduction (version 4.1.1) 119 method and FlowSOM (version 4.1.0)120 clustering approach. The gating strategy is presented in Supplementary Figure 17.
Electroporation efficiency by flow cytometry:
First, a manual data check was performed to ensure the exclusion of technical artifacts and bad-quality samples (clogs, doublets, and dead cells). The unmixing of raw data was performed using single-stain controls as references (no manual compensation was used). Next, doublets were removed, YFP-positive cells were gated manually, and then TRP1 and CD45-positive cells were gated. The gating strategy is presented in Supplementary Figure 18.

☒ Tick this box to confirm that a figure exemplifying the gating strategy is provided in the Supplementary Information.
